# Hyperlactataemia Following Crystalloid Cardiopulmonary Bypass Priming in Paediatric Cardiac Surgery—Benign or Malignant? A Retrospective Study

**DOI:** 10.3390/children11111379

**Published:** 2024-11-13

**Authors:** Philippa Jane Temple Bowers, Michael Daley, Nicole Yvette Renee Shrimpton, Adrian Mattke, Fumiaki Shikata, Kim Betts, Anthony Black, Supreet Prakash Marathe, Prem Venugopal, Nelson Alphonso

**Affiliations:** 1 Department of Cardiac Surgery, Queensland Children’s Hospital, Level 7F Clinical Directorate 501 Stanley Street, Brisbane, QLD 4101, Australia; 2 School of Medicine, University of Queensland, Brisbane, QLD 4072, Australia; 3 Cardiac Surgery (Perfusion), Queensland Children’s Hospital, Brisbane, QLD 4101, Australia; 4 Pediatric Intensive Care Unit, Queensland Children’s Hospital, Brisbane, QLD 4101, Australia; 5 School of Population Health, Curtin University, Perth, WA 6845, Australia

**Keywords:** paediatric cardiac surgery, crystalloid prime, postoperative hyperlactataemia, inotropic support, ICU stay

## Abstract

Background: Various mechanisms leading to early hyperlactataemia post-cardiac surgery have been postulated. Specifically, in the paediatric population, benign early hyperlactataemia may be associated with crystalloid priming in the cardiopulmonary bypass circuit. The aim of this study was to review paediatric patients who had crystalloid prime and assess their outcomes. Methods: A retrospective review of paediatric patients who underwent cardiac surgery with crystalloid prime at our institution between November 2014 and May 2018 was performed. Data were collected from medical and laboratory records. Results: Among 569 patients, 237 (42%) received a crystalloid prime; 51 (22%) were excluded due to intraoperative hyperlactataemia. Of the remaining 186 patients, 98 (53%) developed hyperlactataemia postoperatively. Patients with hyperlactataemia had longer cardiopulmonary bypass and aortic cross-clamp times but similar Aristotle complexity scores. Patients with postoperative hyperlactataemia had higher peak VIS [median 8 (IQR 0–8) vs. 5 (IQR 0–8)] within the first 24 h (*p* = 0.002). However, there was no difference in the duration of ventilation between the two groups (*p* = 0.14). Yet only 58% of patients with hyperlactataemia were discharged from the ICU within 24 h, compared to 78% without hyperlactataemia. Conclusions: In this study population, transient postoperative hyperlactataemia in paediatric patients with crystalloid prime may not necessarily indicate tissue hypoxaemia. Despite a similar duration of ventilation in patients with and without hyperlactataemia, patients with hyperlactataemia had a longer duration of inotropes and ICU stay. Consideration should be given to discontinuing inotropes in patients with crystalloid prime and postoperative early hyperlactataemia once they are extubated.

## 1. Introduction

Lactate production results from glycolysis under anaerobic conditions by the conversion of pyruvate to lactate-by-lactate dehydrogenase [1]. The serum concentration of lactate is an important marker for tissue oxygenation and is one of the laboratory parameters closely monitored after cardiac surgery [2,3]. Elevated lactate concentrations may reflect an imbalance between oxygen delivery and consumption but may also occur without poor tissue oxygenation. Cohen and Woods previously classified lactic acidosis into Type A, with clinical evidence of tissue hypoperfusion, or Type B, with no clinical evidence of tissue hypoperfusion [4]. The impact of the cardiopulmonary bypass prime solution on on-pump acid–base balance and lactate levels in adults has previously been studied without clear conclusions [5,6].

As such, regular monitoring of lactate levels has often been used in the intensive care unit (ICU) setting as a surrogate maker of end-organ perfusion and to further direct inotropic support in critically ill patients [2].

Within the paediatric population, neonates, infants and small children often undergo cardiopulmonary bypass with a blood prime to reduce the effects of haemodilution. In older children with a bloodless cardiopulmonary bypass (CPB) prime, it has previously been suggested that elevated lactate levels may not necessarily indicate a state of tissue hypoperfusion [7,8].

This study aimed to review children with a crystalloid prime and assess their outcomes and postoperative course.

## 2. Materials and Methods

Patient and perioperative data were collected retrospectively from medical and laboratory records at the Queensland Children’s Hospital, Brisbane, from November 2014 to May 2018. The variables collected included descriptors about the patient and disease, cardiopulmonary bypass, postoperative course and laboratory parameters. In the latter part of the study, pyruvate levels and the pyruvate/lactate ratio were also recorded.

Ethics approval was obtained (LNR/QCHQ/44329), and the need for consent was waived.

The study included all patients who underwent cardiac surgery using cardiopulmonary bypass during the study period. From this group, we identified patients with a crystalloid prime of their cardiopulmonary bypass circuit. Hyperlactataemia was defined as serum lactate concentration >2.0 mmol/L. Vasoinotropic scores were calculated as previously described by Gaies et al. [9]. Crystalloid bloodless cardiopulmonary bypass priming was performed using Plasma-Lyte^®^ (Baxter AHB2534, Australia), sodium bicarbonate (8.4% injection 8.4 gm in 100 mL), heparin (5000IU in 5 mL), calcium chloride (10% injection 1 gm in 10 mL) and 20% human albumin. No exogenous lactate was added to the patient’s cardiopulmonary bypass prime. Plasma-lyte^®^ contains acetate. Lactate is not a constituent of Plasma-lyte^®^. Plasma-lyte^®^ was also used for zero-balance ultrafiltration (maximum volume 1000 mL). Patients who demonstrated intraoperative hyperlactataemia on the post-modified ultrafiltration arterial blood gas in the operating theatre were excluded from the analysis.

### Statistical Analysis

Analyses were performed in Stata version 14 (StataCorp., College Station, TX, USA) and R version 3.5.1 software (R Foundation, Vienna, Austria, http://www.r-project.org, accessed on 3 April 2020. Unless otherwise stated, values are given as mean ± standard deviation for normally distributed data or median (IQR) for skewed data. Statistical significance was defined as *p* < 0.05. Comparison of groups was performed using the two-sample *t*-test or Wilcoxon rank-sum test.

## 3. Results

**Figure 1:**
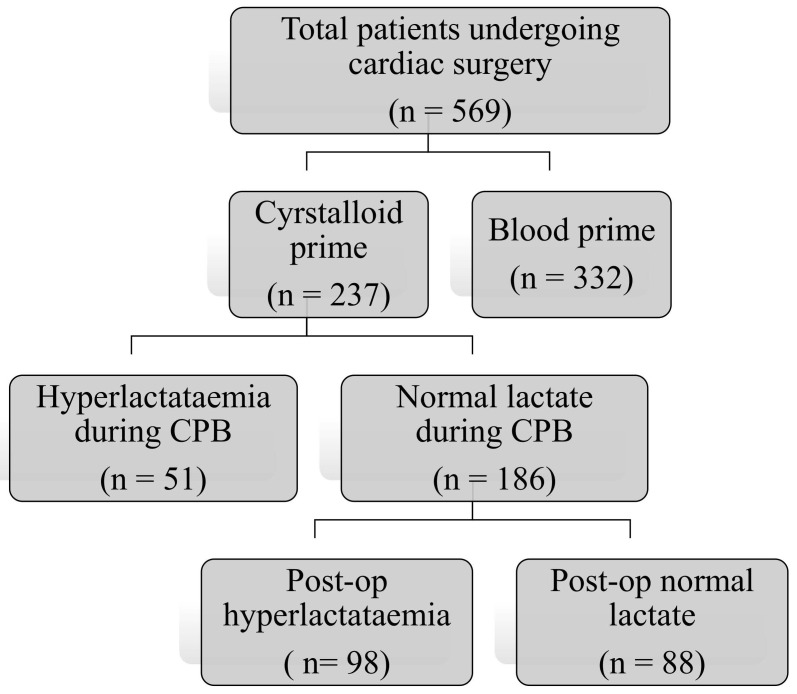
Study flow diagram.

Five hundred sixty-nine patients underwent cardiac surgery using cardiopulmonary bypass during the study period. Of these, 237 (42%) patients had their cardiopulmonary bypass circuit primed with the crystalloid prime solution. Fifty-one (22%) patients were noted to have hyperlactataemia after modified ultrafiltration. They were excluded, leaving 186 patients with a normal lactate level after modified ultrafiltration for analysis. Of these 186 patients, 98 (53%) developed hyperlactataemia during the postoperative period (Figure 1).

Table 1 describes the basic demographics of the study population. No patient required postoperative ECMO. Two patients underwent a period of deep hypothermic circulatory arrest, one patient underwent delayed sternal closure and one patient underwent mediastinal exploration for bleeding. Additionally, three patients (two with hyperlactataemia) developed postoperative pericardial effusions requiring drainage.

Patients who developed hyperlactataemia had longer cardiopulmonary bypass and aortic cross-clamp times (*p* = 0.03 and *p* = 0.02, respectively). However, there was no difference in the Aristotle complexity score between those who developed hyperlactataemia (median 8.8, IQR: 5.5–10.5) and those who did not (median 7.5, IQR: 4–11) (*p* = 0.47).

In patients with postoperative hyperlactataemia, the median peak lactate was 3.0 mmol/L (IQR: 2.4–4.2) and the median time to peak lactate was 10.5 h (IQR: 8.0–17.3) following termination of cardiopulmonary bypass/modified ultrafiltration.

Pyruvate levels were available for four patients (0.29, 0.5, 0.57 and 0.58 mmol/L, respectively). The lactate to pyruvate ratio was 12, 14, 20 and 26 for the four patients, respectively.

### 3.1. Blood Gas Parameters at Peak Lactate Levels in Patients with Hyperlactataemia

Table 2 provides mean blood gas parameters at peak lactate for patients with hyperlactataemia. These patients were not anaemic (Hb 115.7 ± 24.4) and had no evidence of hypoxaemia (as evidenced by normal mean pO_2_ levels). There was a trend towards an increase in the blood glucose concentration in patients with postoperative elevated lactate levels. However, no patient required treatment for an elevated blood glucose level.

### 3.2. Vasoinotropic Score (VIS)

One hundred and twenty-three patients (66%) required inotropic support during the first 24 h postoperatively. Most patients were supported only with milrinone (99/123, 80%) and/or dopamine (83/123, 67%). The median peak VIS in patients discharged from the ICU within 24 h was 4.5 (IQR: 0–8). Patients with postoperative hyperlactataemia had higher peak VIS [median 8 (IQR 0–8) vs. 5 (IQR 0–8)] within the first 24 h (*p* = 0.002). However, in patients who were discharged within 24 h, there was no difference in the peak VIS between patients with postoperative hyperlactataemia (median: 5, IQR: 0–10) and those with normal lactate (median: 2, IQR: 0–7, *p* = 0.15).

### 3.3. Discharge from the Intensive Care Unit (ICU)

A total of 4 (2%) patients were discharged from the ICU on the day of surgery, 122 (68%) patients were discharged the following morning and 60 (32%) patients were discharged after two or more days. Patients with postoperative hyperlactataemia had a longer ICU stay (*p* = 0.003).

### 3.4. Patients Discharged from ICU ≤ 24 h Postoperatively

A total of 126 (68%) patients were discharged from the ICU ≤ 24 h postoperatively. Of these, 57 (45%) patients developed hyperlactataemia, which resolved in the first 24 h. There was no difference in the median duration of ventilation time between the two groups of patients [hyperlactataemia 5.8 h (IQR: 3.8–7.8)] compared with 5.2 h (IQR: 3.0–6.5) for patients without hyperlactataemia (*p* = 0.14)]. However, only 58% (n = 57) of patients with hyperlactataemia (n = 98) were discharged within 24 h, as compared to 78% of patients without hyperlactataemia (n = 88), which is summarised in Table 3 below.

### 3.5. Patients Discharged from ICU >24 h Postoperatively

Sixty (32%) patients were discharged from the ICU after 24 h. In this group of patients, the median ICU stay for patients with early hyperlactataemia (n = 41) was three days (IQR 2–4) compared to a median of 2 days (IQR 2–3) for patients without hyperlactataemia (n = 19) (*p* = 0.051). Of these 60 patients, 68% had postoperative hyperlactataemia (n = 41), compared with the 32% of patients without hyperlactataemia (n = 19, *p* < 0.004).

## 4. Discussion

We reviewed patients undergoing cardiac surgery with crystalloid priming of their cardiopulmonary bypass circuit with a focus on those who developed high early postoperative lactate levels (<24 h). Our study demonstrated that early postoperative hyperlactataemia was associated with longer CPB and aortic cross-clamp times despite similar Aristotle complexity scores. Even though the difference in Aristotle score did not reach statistical significance, the patients who developed postoperative hyperlactataemia had higher Aristotle scores. The median duration of ventilation was similar in both groups (<6 h). However, only 58% of patients were discharged within 24 h, compared to 80% of patients who did not develop early postoperative hyperlactataemia.

Overall, early postoperative hyperlactataemia was associated with longer cardiopulmonary bypass and aortic cross-clamp times. This may have contributed to the raised lactate levels as these patients also required a longer duration of inotropic support (albeit with milrinone and/or dopamine) in the ICU.

However, hyperlactataemia does not necessarily translate to worse early postoperative outcomes. The median duration of ventilation was similar in both groups (<6 h), implying that the patients with hyperlactataemia were haemodynamically stable despite the raised lactate levels. There was no difference in the VIS score in patients discharged from the ICU within 24 h. This is not surprising because, in our hospital, patients cannot be discharged to the ward on any inotropes. However, only 60% of patients who developed early postoperative hyperlactataemia were discharged within 24 h despite a similarly short duration of ventilation. There was no evidence of tissue hypoxia in the remaining 40% of patients who developed early postoperative hyperlactataemia. Again, this supports the suggestion that transient postoperative hyperlactataemia is benign in an otherwise clinically stable paediatric patient with a crystalloid prime during CPB. The most likely explanation for the higher VIS score in this group of patients is that they were kept on low-dose inotropes overnight because of the hyperlactataemia despite being extubated.

In clinical practice, any prolonged ICU stay has a significant negative impact on patient flow. Based on the findings in our study, consideration should be given to discontinuing inotropes in patients with a crystalloid prime and postoperative early hyperlactataemia once they are deemed suitable for extubation. These patients can then promptly progress to discharge from the ICU.

In patients discharged from the ICU after 24 h, the median ICU stay for patients with early hyperlactataemia was longer (a median of 3 days) compared to a median of 2 days for patients without hyperlactataemia. Other clinical factors likely contributed to the timing of discharge from the ICU in this group of patients.

One additional finding of note in our study is that patients with hyperlactataemia postoperatively had a trend to a raised glucose level compared to those who did not develop hyperlactataemia. The reasons for this are unclear. Previous studies have suggested that postoperative hyperlactataemia is benign due to metabolic uncoupling associated with clear prime [7,10].

In our unit, Plasma-lyte^®^ is used during the priming of the cardiopulmonary bypass circuit and ultrafiltration. Plasma-lyte^®^ does not contain lactate and no exogenous lactate was added to the study patients intraoperatively. Furthermore, we monitored lactate levels and electrolyte levels in the cardiopulmonary bypass circuit prime before the institution of cardiopulmonary bypass, and the prime lactate level in any circuit did not exceed 1 mmol/L. Finally, the initial serum lactate level on admission to the ICU was low in every patient, further confirming that exogenous lactate could not have been the reason for the elevated serum lactate levels measured later during the ICU stay.

Pyruvate and the lactate-to-pyruvate ratio (LPR) were recorded for only four patients, all showing LPRs between 12 and 26. An LPR over 25 is associated with tissue hypoxia during circulatory shock [11]. Conversely, lactate elevation that is associated with lower LPRs may indicate a lactate rise that is not related to tissue hypoxia. The oxygen extraction ratio in our patients, along with the low arteriovenous pCO_2_ difference, suggests sufficient tissue oxygen delivery. Lactic acid homeostasis in humans is a complex process [12]. Lactate production as a fuel in humans depends mainly on glycolysis. Under tissue hypoxia conditions, the tricarboxylic acid (TCA) cycle may be inhibited, leading to a reduction in ATP production and activation of both glycolysis and gluconeogenesis pathways. Increased glucose in the cytoplasm, in turn, increases pyruvate, as glucose can be converted to pyruvate through catalytic conversion reactions [12]. Pyruvate, without entering mitochondria, is converted by lactate dehydrogenase to lactate. These metabolic pathways may explain the findings of our study. We speculate that initial (relative) tissue hypoxaemia during CPB, possibly due to a crystalloid prime, leads to TCA cycle inhibition, which in turn leads to increased glucose metabolism, which subsequently leads to an increase in both lactate and pyruvate. Palermo et al. described “metabolic uncoupling” following surgery using CPB, with increased blood sugar levels in patients with type B hyperlactataemia [10]. In that study, insulin treatment did not lead to a shorter duration of lactate increase. While our reasoning about a TCA cycle inhibition, with an increase in cytosolic glucose leading to increased pyruvate concentrations and subsequent lactate conversion, may be somewhat speculative, it matches Palermo’s finding—increased blood sugar levels are a response to TCA cycle slowdown. Insulin treatment will likely not affect the described pathways other than normalising blood glucose levels. To explain this metabolic pathway further, urinary organic acids could be measured during the period of hyperlactataemia, and if intermediate products of the TCA cycle (such as maleate, fumarate or oxaloacetate) are found to be elevated, this could provide support for the hypotheses described above [13].

This study carries the usual limitations of data collection in retrospective reviews of medical records. Furthermore, it remains uncertain if our findings can be generalised, given that it was a single-centre study. Further research, including a larger, multicentre, prospective study would be required to confirm the safety and efficacy of the proposed management strategies. Patients recovering from cardiac surgery are in a complex physiological state, particularly within the first 24 h, with multiple complex variables interacting with each other. Although our study has attempted to account for common variables, unaccounted variables may have impacted early postoperative serum lactate levels.

## 5. Conclusions

In children undergoing cardiac surgery with crystalloid priming of the cardiopulmonary bypass circuit, approximately 50% appear to develop transient hyperlactataemia postoperatively without evidence of tissue hypoxaemia. The transient hyperlactataemia did not result in a prolongation of the duration of ventilation but appeared to prolong the duration of inotropes and impacted early discharge from the ICU (within 24 h). Based on the findings in our study, consideration should be given to discontinuing inotropes in otherwise clinically stable patients with crystalloid prime and postoperative early hyperlactataemia once they are extubated.

## Figures and Tables

**Table 1:** Patient demographics (*n* = 186).

Demographic	Total	Hyperlactataemia Postoperatively (*n* = 98)	Normal Lactate Postoperatively (*n* = 88)	*p*-Value
Female, *n* (%)	82 (44)	38 (39)	44 (50)	0.12
Age at surgery, median	7.9 (4.6–14.3)	8.5 (6.0–12.8)	6.5 (3.7–12.7)	0.01
Body surface area, median (IQR)	0.90 (0.70–1.40)	0.94 (0.78–1.37)	0.81 (0.66–1.41)	0.18
Syndrome, *n* (%)	22 (12)	15 (15)	7 (8)	0.12
Redo sternotomy, *n* (%)	55 (30)	27 (28)	28 (32)	0.52
Aristotle score, median (IQR)	9 (IQR 6.4–11)	8.8 (IQR: 5.5–10.5)	7.5 (IQR: 4–11)	0.47
Cardiopulmonary bypass time in minutes, median (IQR)	66 (44–118)	81 (54–149)	54 (39–86)	<0.001
Aortic cross-clamp time in minutes, median (IQR)	30 (6–70)	41 (12–115)	24 (6–48)	0.004
Vasoinotropic score maximum in 1st 24 h, median (IQR)	6 (0–10)	8 (0–11)	5 (0–8)	0.002
ICU stay ≤ 1 day, *n* (%)	126 (68)	57 (45)	69 (55)	-
ICU stay > 1 day, *n* (%)	60 (32)	41 (68)	19 (32)	0.003
Ventilation time, hours (IQR)	-	5.8 (3.8–7.8)	5.2 (3.0–6.5)	0.14

**Table 2:** Mean arterial blood gas values at the time of peak lactate levels.

Parameter	Value
pH	7.32 ± 0.05
pCO_2_	40.9 ± 6.0
pO_2_	117 ± 39.7
Hb	114.1 ± 22.4
Na^+^	139 ± 2.8
K^+^	4.0 ± 0.6
Cl^−^	106 ± 3.1
HCO_3_^−^	20.6 ± 2.7
Base Excess	−4.7 ± 2.8
Glucose	9.4 ± 2.6
Lactate, median (IQR)	3.7 (2.4–4.1)
Ca^2+^	1.2 ± 0.1
Creatinine	49.1 ± 18.3

**Table 3:** Patients discharged from ICU ≤ 24 h postoperatively.

Demographic	Total	Hyperlactataemia Postoperatively (*n* = 57)	Normal Lactate Postoperatively (*n* = 69)	*p*-Value
Aristotle score, mean ± SD	7.9 ± 3.6	8.2 ± 3.7	7.7 ±3.5	0.45
Cardiopulmonary bypass time, median (IQR)	57 (41–86)	66 (48–99)	54 (39–74)	0.04
Aortic cross-clamp time, median (IQR)	24.5 (5–48)	36 (16–57)	20 (0–39)	0.03
Vasoinotropic score maximum in 1st 24 h, median (IQR)	4.5 (0–8)	5 (0–10)	2 (0–8)	0.15

## Data Availability

All relevant data is included in the manuscript.
